# Supplementary material for: Reviews on common objectives and evaluation indicators for risk communication activities from 2011 to 2017
Source: PeerJ. 2020 Aug 25;8:e9730. doi: 10.7717/peerj.9730 (PMC7453920; doi:10.7717/peerj.9730)
Supplement: Supplemental Information 3 [file peerj-08-9730-s003.docx]

**Table S3. Results of statistical analyses on the association of objective/evaluation indicator with study field, intervention timing, target audience, and communication type.**

|  | | Objective/evaluation indicator | | | | | | |
| --- | --- | --- | --- | --- | --- | --- | --- | --- |
|  |  | Knowledge  increase | Communication satisfaction | Change in risk perception and concern alleviation | Reduction in other psychological distress | Trust  building | Decision making and behavior change | Self-efficacy improvement |
|  |  | *n* (%) | *n* (%) | *n* (%) | *n* (%) | *n* (%) | *n* (%) | *n* (%) |
| Study field | Medicine (*n* = 169) | 73 (43) | 33 (20) | 66 (39) | 5 (3) | 10 (6)^*;^ *^a^* | 105 (62) | 9 (5) |
|  | Food safety (*n =* 16) | 6 (38) | 5 (31) | 8 (50) | 0 (0) | 2 (13)^*;^ *^a^* | 7 (44) | 0 (0) |
|  | Chemical substances (*n* = 13) | 7 (54) | 1 (8) | 7 (54) | 1 (8) | 0 (0)^*;^ *^a^* | 9 (69) | 0 (0) |
|  | Other disasters/  emergencies (*n* = 22) | 6(27) | 1 (5) | 13 (59) | 0 (0) | 5 (23)^*;^ *^a^* | 13 (59) | 0 (0) |
| Intervention timing | Pre-crisis (*n* = 250) | 99 (40) | 40 (16) | 119 (48)^*^ | 6 (2) | 17 (7) | 152 (61) | 11 (4) |
|  | Crisis (*n* = 10) | 2 (20) | 1 (10) | 1 (10)^*^ | 0 (0) | 2 (20) | 5 (50) | 0 (0) |
| Target audience | Citizens/NPOs (*n* = 240) | 96 (40) | 42 (18) | 113 (47) | 5 (2) | 17 (7) | 145 (60) | 11 (5) |
|  | Other (*n* = 25) | 9 (36) | 1 (4) | 9 (36) | 1 (4) | 2 (8) | 14 (56) | 0 (0) |
| Communication type | Individual/ small group communication (*n* = 90) | 38 (42) | 16 (18) | 32 (36)^*^ | 4 (4) | 5 (6) | 63 (70)^*^ | 3 (3) |
|  | Large group/  mass communication (*n* = 175) | 67 (38) | 27 (15) | 90 (51)^*^ | 2 (1) | 14 (8) | 96 (55)^*^ | 8 (5) |

This table includes only studies that did not belong to multiple categories in the variables of study field, intervention timing, target audience, and communication type. Percentages are based on the total number of each value.

* Pearson’s chi-squared test or Fisher’s exact test *P* < 0.05.

*a, b* Results of multiple comparisons based on Fisher’s exact test. Significant differences lie between groups with a single “*a*” and ones with a single “*b*” (*P* < 0.05).
